# Multiparametric MRI dataset for susceptibility-based radiomic feature extraction and analysis

**DOI:** 10.1038/s41597-024-03418-6

**Published:** 2024-06-04

**Authors:** Cristiana Fiscone, Giovanni Sighinolfi, David Neil Manners, Lorenzo Motta, Greta Venturi, Ivan Panzera, Fulvio Zaccagna, Leonardo Rundo, Alessandra Lugaresi, Raffaele Lodi, Caterina Tonon, Mauro Castelli

**Affiliations:** 1 Department of Biomedical and Neuromotor Sciences, University of Bologna, Bologna, Italy; 2 Functional and Molecular Neuroimaging Unit, IRCCS Istituto delle Scienze Neurologiche di Bologna, Bologna, Italy; 3 Department for Life Quality Sciences, University of Bologna, Bologna, Italy; 4 UOSI Riabilitazione Sclerosi Multipla, IRCCS Istituto delle Scienze Neurologiche di Bologna, Bologna, Italy; 5 Department of Imaging, Cambridge University Hospitals NHS Foundation Trust, Cambridge Biomedical Campus, Cambridge, United Kingdom; 6 Department of Radiology, University of Cambridge, Cambridge, United Kingdom; 7 Investigative Medicine Division, Radcliffe Department of Medicine, University of Oxford, Oxford, United Kingdom; 8 Department of Information and Electrical Engineering and Applied Mathematics, University of Salerno, Fisciano, Italy; 9 NOVA Information Management School (NOVA IMS), Universidade NOVA de Lisboa, Campus de Campolide, 1070-312 Lisbon, Portugal

**Keywords:** Biomarkers, Multiple sclerosis

## Abstract

Multiple sclerosis (MS) is a progressive demyelinating disease impacting the central nervous system. Conventional Magnetic Resonance Imaging (MRI) techniques (e.g., T_2_w images) help diagnose MS, although they sometimes reveal non-specific lesions. Quantitative MRI techniques are capable of quantifying imaging biomarkers *in vivo*, offering the potential to identify specific signs related to pre-clinical inflammation. Among those techniques, Quantitative Susceptibility Mapping (QSM) is particularly useful for studying processes that influence the magnetic properties of brain tissue, such as alterations in myelin concentration. Because of its intrinsic quantitative nature, it is particularly well-suited to be analyzed through radiomics, including techniques that extract a high number of complex and multi-dimensional features from radiological images. The dataset presented in this work provides information about normal-appearing white matter (NAWM) in a cohort of MS patients and healthy controls. It includes QSM-based radiomic features from NAWM and its tracts, and MR sequences necessary to implement the pipeline: T_1_w, T_2_w, QSM, DWI. The workflow is outlined in this article, along with an application showing feature reliability assessment.

## Background & Summary

Quantitative Susceptibility Mapping (QSM)^1,2^ is an advanced Magnetic Resonance Imaging (MRI) technique used to quantify and map the magnetic susceptibility (χ) of various structures within the body, with a primary focus on the brain. This technique is frequently employed to investigate and visualize medical conditions that alter the magnetic properties of bodily tissues, such as the presence of iron deposits, hemorrhages, or de-myelinating processes. Since the early 2010s, demand for more precise and quantitative assessments of magnetic tissue properties has driven interest in the application of QSM in neuroimaging.

The development of QSM builds on previous research in MRI and χ-based techniques. In fact, numerous neurological conditions in both adults and children have demonstrated abnormal accumulations of blood-related substances or mineral deposits. Thus, Susceptibility-Based Imaging (SBI)^3^ has found application in clinical settings. While traditional SBI methods, like Susceptibility Weighted Imaging (SWI)^4,5^, offer valuable insights, they come with certain limitations compared to QSM^2^, which provides quantitative measurements, enables comparisons within and between different groups, and facilitates the discrimination between diamagnetic and paramagnetic substances.

One of the most investigated applications of QSM is the examination of neurodegenerative and neuroinflammatory disorders^6^, including Multiple Sclerosis (MS), which is an autoimmune demyelinating disease affecting the Central Nervous System (CNS) and presenting a wide array of symptoms such as fatigue, motor or sensory loss in limbs, cognitive decline, and visual disturbances^7^. The current diagnosis of MS relies on the McDonald criteria, which combine clinical observations, laboratory biomarkers, and imaging data^8^. Traditional MRI has played a crucial role in these criteria and is widely used to monitor disease progression. Nevertheless, conventional MRI techniques reveal established lesions without providing detailed insights into the underlying mechanisms responsible for demyelination^9^: focal or confluent white matter (WM) alterations, which are found in over 95% of MS patients, can be detected, but the presence of these lesions is not an absolute requirement for diagnosing the disease, as non-specific WM lesions can also occur in healthy individuals over 50 years old or those affected by other neurological diseases.

The development of innovative quantitative MRI (qMRI) techniques capable of quantifying imaging biomarkers *in vivo* holds promise for exploring the microstructure of the brain^10^ and its metabolic processes^11^ to unravel the pathophysiology of MS and potentially reveal signs of pre-clinical inflammatory demyelination. QSM, as a qMRI technique, may aid in the investigation of both damaged and undamaged brain tissue in patients with MS^12–15^.

In the last twenty years, radiomics has emerged as a quantitative analytical tool for personalized medicine using medical imaging^16,17^. It includes a set of techniques that extract complex and multi-dimensional features from radiological images, including characteristics such as intensity histograms and textural patterns within the Region or Volume of Interest (ROI/VOI) analyzed^18^. Since its introduction, radiomics has found applications across several medical image modalities. In the field of MR neuroimaging, a significant focus has been directed toward the study of brain tumors, making use of anatomical T_1_– and T_2_–weighted (T_1_w and T_2_w) images for purposes such as tumor characterization and grading^19,20^ or the assessment of treatment response and clinical outcomes^21^.

The dataset^22^ we provide in this work includes QSM-based radiomic features from Normal Appearing White Matter (NAWM) and its tracts, extracted in a mixed group of patients with MS and healthy controls, and all the different MR sequences necessary to implement the pipeline: for each subject, morphological T_1_w and T_2_w, QSM and Diffusion Weighted Imaging (DWI) for Diffusion Tractography Imaging (DTI). An example of an application based on those data^23^ is shown in the last section.

## Methods

### Participants

For this work, data from 100 MS patients were obtained from the repository of the Neuroimaging Laboratory (IRCCS Istituto delle Scienze Neurologiche di Bologna, Bologna, Italy), along with data from 50 healthy control subjects. Inclusion criteria are as follows: patients had to be older than 18 years, with relapsing-remitting, primary progressive or secondary progressive MS course according to the 2017 revision of the McDonald diagnostic criteria, and to receive intravenous therapy with anti-CD20 monoclonal antibodies. Healthy controls had to be older than 18 years old; furthermore, the MRI exam had to be negative for abnormal cerebral atrophy, cortical and subcortical iron accumulation and CSF circulation disturbances. The demographic characteristics of the sample are summarized in Table 1.

**Table 1:** Demographic details of the sample.

	HC	MS
**N**	50	100
**F:M**	31:19	58:42
**Age (y)**	57.1 ± 16.7	47.6 ± 11.4
	(24–86)	(23–73)

Sex and age (mean ± standard deviation), with range in parenthesis, are given for healthy controls and patients with MS.

The data provided are related to scans performed between November 2020 and August 2023, except for one MR sequence for one healthy subject acquired in 2018. Acquisition and processing software and pipelines have basically remained the same throughout the relevant period, guaranteeing the homogeneity of the data used for analysis; small changes that were implemented are illustrated in the following sections.

### Ethical approval and informed consent

Ethical approval for the study was obtained by the Institutional Review Board “Area Vasta Emilia Centro” (AVEC) (approval number AUSLBO 2023/CE 23043); written informed consent was obtained from all participants. Data has been anonymized and de-identified to ensure participating individuals cannot be identified from the information or images provided.

### MRI data acquisition

**Fig. 1:**
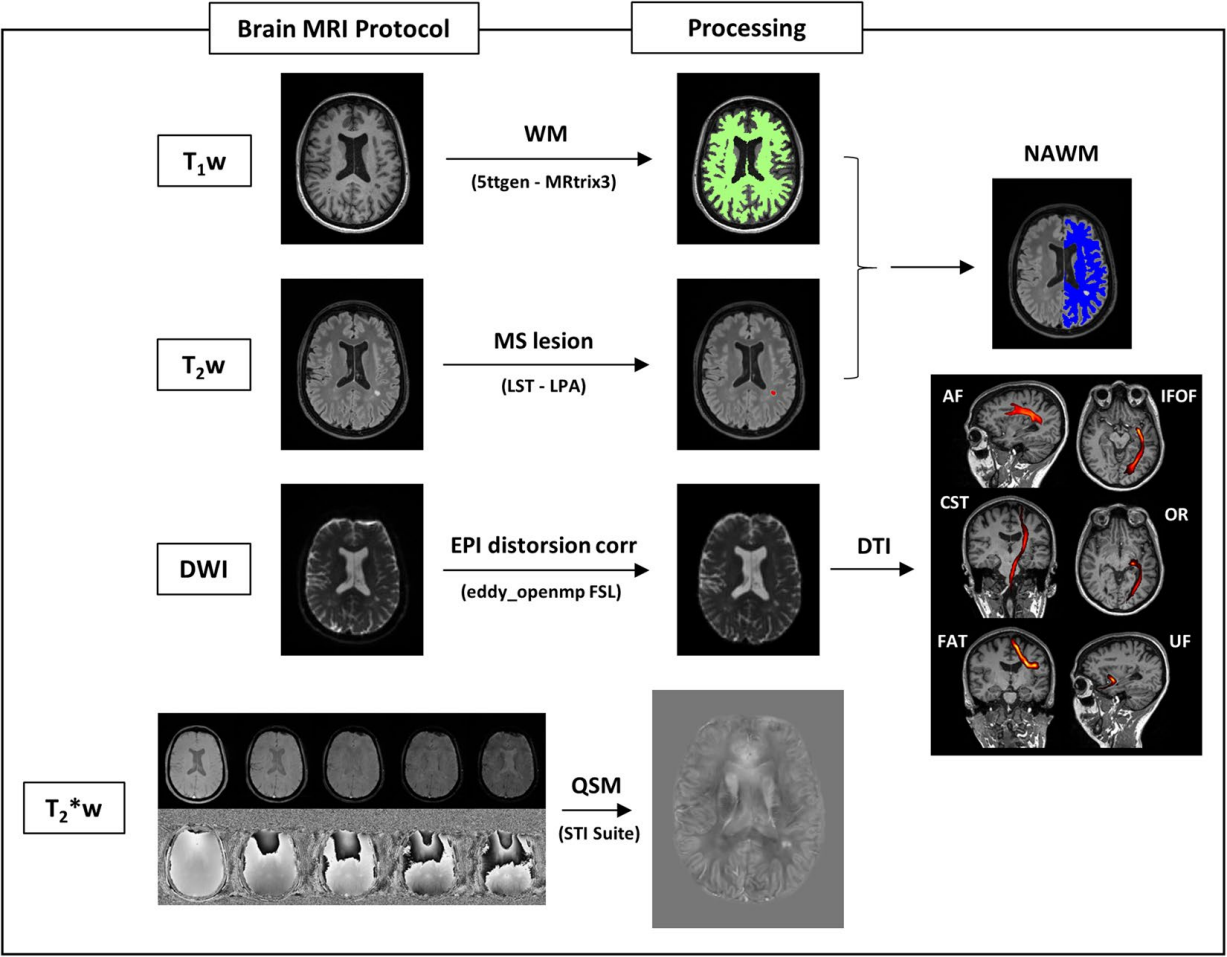
Scheme of the acquisition and processing pipeline. The brain MRI protocol provided: T_1_w, T_2_w, DWI and T_2_*w images (in this figure: patient with MS, F/38 years old): 1) T_1_w images were used to obtain white matter segmentation using 5ttgen from MRtrix3 2) LPA algorithm from FSL was applied to T_2_w images to obtain MS lesion mask. Merging WM segmentation with the MS lesion mask, the normal-appearing-white-matter segmentation mask was obtained. We run LST only on patients with MS; for controls, NAWM corresponds to WM from MRtrix3. 3) DWI images were pre-processed as explained in the specific section and diffusion tractography imaging automatic pipeline was applied, obtaining six white matter tracts (arcuate fasciculus, cortico-spinal tract, frontal aslant tract, inferior frontal-occipital fasciculus, optic radiation, uncinate fasciculus); VOIs from tractography reconstruction were merged with MS lesion mask to exclude damaged tissue. 4) T_2_*w images were processed to obtain QSM reconstructions, as explained in the specific section. All the images/masks were registered in the T_1_w space. Radiomic features were extracted from QSM images in 14 volumes (six white matter tracts and total normal appearing white matter, left and right hemisphere).

Figure 1 shows the workflow for the acquisition and processing pipeline.

Scans were performed on a 3-T clinical scanner (Magnetom Skyra; Siemens Healthineers, Erlangen, Germany) equipped with a whole-body transmit and a 64-channel Head/Neck receiver coil. Sequences included in the MRI protocol and their details are shown in Table 2.

**Table 2:** Details of the sequences included in the MRI protocol.

	MPRAGE	FLAIR	HARDI DWI	QSM
	3D T_1_w	3D SPACE T_2_w	2D EPI single-shot	3D GRE T_2_*w
**Plane**	sagittal	sagittal	axial	axial
**TR (ms)**	2300	5000	4300	53
**TE1 (ms)**	2.98	428	98	9.42
**ΔTE (ms)/n° TEs**	—	—	—	9.42 / 5
**TI (ms)**	900	1800	—	—
**SR (mm**^**3**^**)**	1 × 1 × 1	1 × 1 × 1	2 × 2 × 2	0.5 × 0.5 × 1.5
**FA (°)**	9	120	90	15
**Scan time**	5’21”	5’55”	~9’	8’45”

(TE = echo time, TR = time of repetition, TI = Time of Inversion, FA = Flip Angle, MPRAGE = Magnetization Prepared RApid Gradient Echo, SPACE = Sampling Perfection with Application optimized Contrast using different flip angle Evolution, FLAIR = FLuid Attenuated Inversion Recovery, GRE = GRadient Echo, HARDI = High Angular Resolution Diffusion Imaging, EPI = Echo Planar Imaging).

We modified the DWI sequence during the study, going from single-shell (5 unweighted volumes and 64 volumes with b = 2000 s/mm^2^) to multi-shell (8 unweighted volumes, 12 volumes with b = 300 s/mm^2^, 30 volumes with b = 1000 s/mm^2^ and 64 volumes with b = 2000 s/mm^2^) acquisition. This change should have a negligible effect on tissue contrast for equivalently weighted volumes, given the processing method employed. In the dataset, the type of sequence is indicated for each subject. Sequences were acquired with Anterior-Posterior (AP) phase encoding. An additional sequence with inverted phase encoding (PA) (~ 4’ scan time) was acquired to correct EPI distortion artifacts in the EPI volumes: for single-shell measurements, three unweighted volumes were acquired, while for multi-shell, the sequence was re-acquired by repeating each volume acquisition with weighting 0–1000 s/mm^2^. Concerning tractography reconstructions, we used volumes with a b-value of 2000 s/mm^2^ for single- and multi- shell measurements without changing the pipeline. The extraction of radiomics features uses only the volumes of the reconstructed tracts, consistent between the two sequences. Raw data of unweighted and b = 2000 s/mm^2^ volumes are available in the dataset^22^.

To qualify for inclusion in the dataset, complete and artifact-free MRI acquisition was essential for both patient and healthy control groups. Each examination comprises all the sequences (MPRAGE, FLAIR, DWI with both AP and PA phase encoding, QSM) necessary for the processing pipeline. The images were assessed to ensure they were of sufficient quality, devoid of significant motion artifacts or other distortions, to make them suitable for analysis using the standard pipeline.

MR sequences were derived from the same exam for all but three healthy subjects (subj-006 [F/57yo], subj-007 [F/46yo], and subj-040 [M/39yo]). In each of these instances, the FLAIR image has been acquired during a prior examination, approximately 30, 5, and 42 months before, respectively. For consistency, we include the FLAIR sequence for all the subjects, both patients and controls, even though it was used only to automatically segment patients’ MS lesions; in this context, it was not used to analyze the healthy controls. Considering this and the fact that subj-006, subj-007 and subj-040 were healthy and not elderly, the mismatch between the days of the MRI exams does not affect either the pipeline or the extracted features.

### MRI data processing

Raw DICOM data were converted to NIfTI format using dcm2niix (https://github.com/rordenlab/dcm2niix). Over the years, the software version changed from v1.0.20171215 to v1.0.20210317. This change does not affect recorded pixel intensities.

#### Morphological T_1_w and T_2_w processing

Original morphological images T_1_w and T_2_w were used without further processing; T_2_w images were linearly registered onto the corresponding T_1_w using Functional Magnetic Resonance Imaging of the Brain (FMRIB) Software Library (FSL)^24^ (v. 6.0.4) (FSL’s Linear Image Registration Tool [FLIRT]^25,26^). All inter-modality registrations were performed using 12 degrees of freedom (affine transformations).

#### QSM processing

To obtain χ maps, phase maps from the five echo times were processed individually by Laplacian unwrapping^27^ and Variable kernel Sophisticated Harmonic Artifact Reduction for Phase data (V-SHARP) as background field removal^28^. The processing requires a brain binary mask obtained by skull-stripping the magnitude image of the first echo time using the FSL^24^ Brain Extraction Tool (BET)^29^.

Multi-echo phase data were combined into a single-phase image through a weighted sum over echoes of processed phase maps, using as weights^30^:

$$w\left(T{E}_{i}\right)=\frac{T{E}_{i}{e}^{-T{E}_{i}/{T}_{2}^{* }}}{{\sum }_{j=1}^{5}T{E}_{j}{e}^{-T{E}_{j}/{T}_{2}^{* }}}$$

The iterative least square (iLSQR) technique was used as a dipole inversion method^31^. STI Suite^32^ was used for the processing. The resulting QSM image was linearly registered using FLIRT^25,26^ to the corresponding morphological T_1_w image.

Cerebrospinal Fluid (CSF) served as the reference tissue, employing the *zero-referencing* method^33^. This choice is widely adopted because CSF susceptibility values do not show changes related to the subject’s age or pathological condition, and orientation dependence can be excluded. However, since the sizes of the ventricles vary and the CSF signal often appears non-uniform in QSM reconstructions, we did not consider the entire ventricles but three small volumes instead (atrium, anterior horns, and central part), as suggested by Straub and colleagues^34^. An original atlas-based method was implemented to automatically identify the three volumes on each exam. Isotropic 1-mm MPRAGE maps of 60 subjects, both healthy controls and patients, were non-linearly registered (elastic registration) using FSL^24^ FNIRT (https://fsl.fmrib.ox.ac.uk/fsl/fslwiki/FNIRT), after brain extraction using BET^29^, onto the space of a selected reference subject. The deformation matrix returned was combined with the linear transformation mapping QSM to T_1_w in the same exam so that all 60 QSM-magnitude maps were averaged in the same space.

Three small bilateral CSF volumes were manually defined on three adjacent slices of the atlas. The volumes were back-registered onto the original QSM map for each subject and the mean susceptibility value was calculated to establish the reference.

Motion artifacts pose a significant challenge for QSM due to its reliance on phase information extracted from MRI data. Even minor movements during image acquisition can introduce errors in phase consistency across slices or time frames, undermining the accuracy of susceptibility calculations. Notably, the QSM sequence is typically lengthy - lasting approximately 9 minutes in the current study -, allowing a lot of time for artifacts to occur. These artifacts manifest as geometric distortion, ghosting effects, and signal loss, ultimately compromising the quality and reliability of QSM images. To address this issue, rigorous quality control is essential. It was implemented by evaluating each QSM reconstruction individually.

**Fig. 2:**
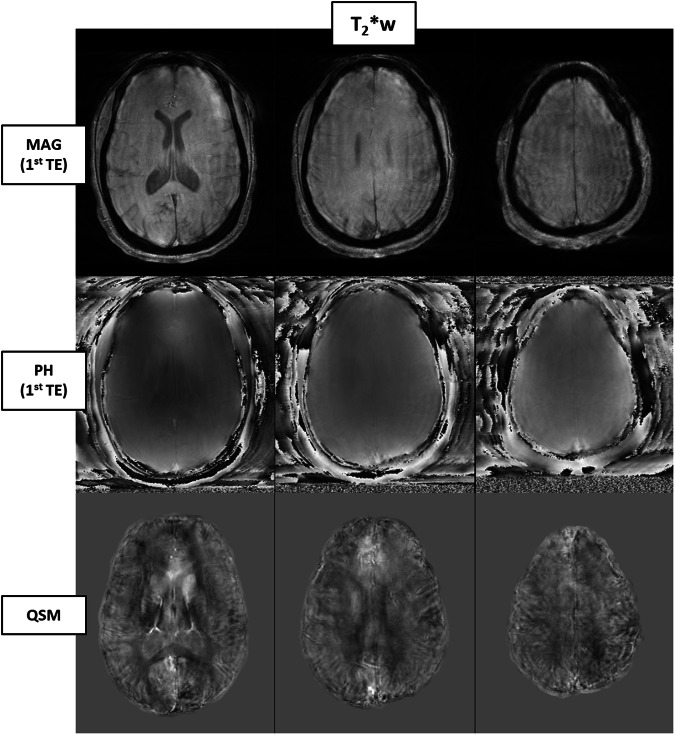
Example of one exam (patient with MS, F/29 years old) excluded because of the quality of QSM image, showing movement artifacts and not considered suitable for the analysis: in the first and second row, magnitude and phase raw data from the first echo time; in the third row, the QSM reconstruction.

Figure 2 shows an example of a QSM reconstruction excluded from the study due to motion artifacts. In this instance, the presence of motion was apparent even in the raw magnitude maps, facilitating the decision to exclude directly after the acquisition. However, QSM images are derived from phase data, which may not exhibit such evident artifacts in their raw form because of the discontinuities present before the unwrapping stage. Such artifacts can remain undetected until post-processing is performed.

#### DWI processing

An automated diffusion and tractography processing pipeline was implemented as previously described^35,36^. DWI images underwent a process of skull-stripping using BET^29^. They were denoised using the **dwidenoise** function from MRtrix3^37^ (v. 3.0.2) with a principal component analysis approach. To address susceptibility-related distortion, we employed the FSL^24^ function **topup**. Subsequently, we addressed susceptibility effects, eddy currents, and signal dropout using the FSL^24^ **eddy_openmp** function^38^. Linear registration between diffusion measurements and T_1_-weighted images was conducted using the **epi_reg** function from FLIRT^25,26^. Diffusivity was modeled along the spatial eigenvector using the tensor model and a high-order fiber modeling technique. Moreover, we adopted a probabilistic streamline approach to evaluate crossing fibers.

Diffusion images were used to estimate fiber orientation distributions by the single tissue, single shell spherical deconvolution algorithm ‘csd’ implemented by the **dwi2fod** function^39^ from MRtrix3. The Diffusion Tractography pipeline is described in the section VOI segmentation. In total, six WM tracts were defined: arcuate fasciculus (AF), cortico-spinal tract (CST), frontal aslant tract (FAT), inferior frontal-occipital fasciculus (IFOF), optic radiation (OR), and uncinate fasciculus (UF).

### VOI segmentation

**Fig. 3:**
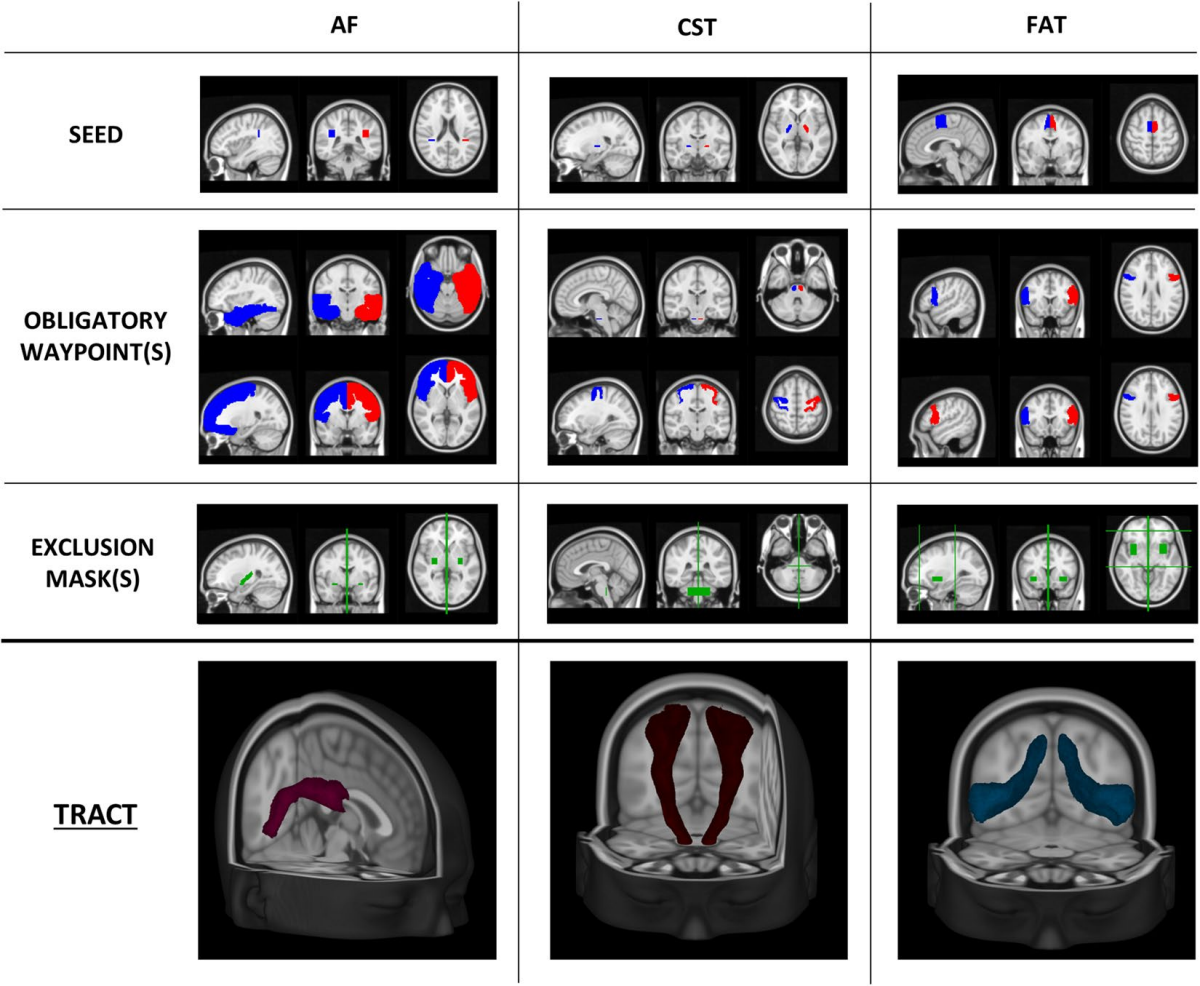
Seed, inclusion and exclusion regions for AF, CST and FAT. Regions were defined on the MNI152 standard brain and used for streamlining generation and selection to reconstruct WM tracts. Region descriptions are in Table 2. In the last row, the appearance of each tract reconstruction is shown in the MNI152 space, obtained by averaging tracts of 30 healthy controls (AF = Arcuate Fasciculus, CST = Cortico-Spinal Tract, FAT = Frontal Aslant Tract, OB WAY = Obligatory Waypoint(s), Excl = Exclusion mask(s), MNI152 = Montreal Neurological Institute’s 152, WM = White Matter).

**Fig. 4:**
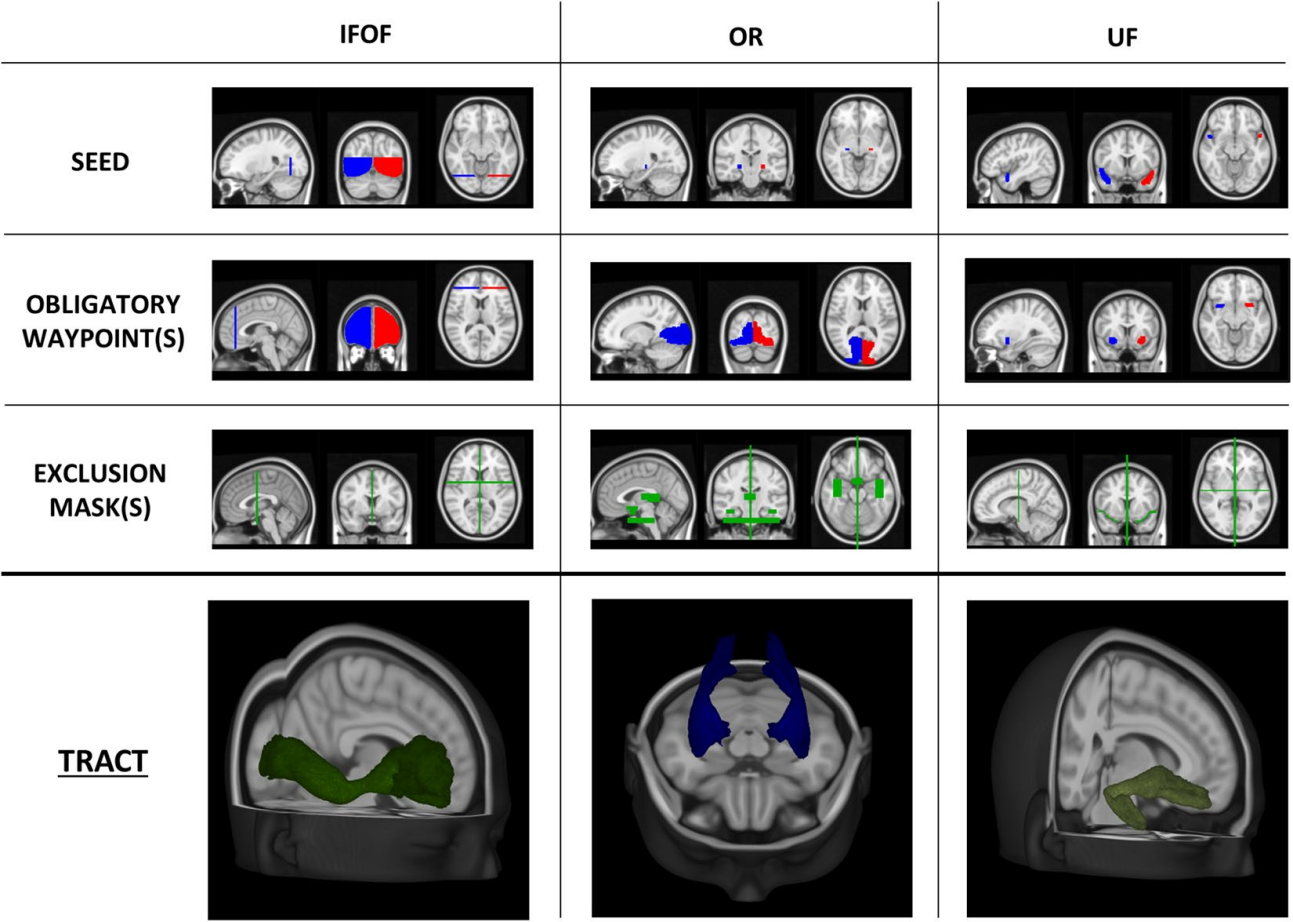
Seed, inclusion and exclusion regions for IFOF, OR and UF. Regions were defined on the MNI152 standard brain and used for streamlining generation and selection to reconstruct WM tracts. Region descriptions are in Table 2. In the last row, the appearance of each tract reconstruction is shown in the MNI152 space, obtained by averaging tracts of 30 healthy controls (IFOF = Inferior Frontal-Occipital Fasciculus, OR = Optic Radiation, UF = Uncinate fasciculus, OB WAY = Obligatory Waypoint(s), Excl = Exclusion mask(s), MNI152 = Montreal Neurological Institute’s 152, WM = White Matter).

VOIs selected for feature extraction were segmented as follows:

- White matter (WM): WM segmentation was conducted using the MRtrix3 tool 5ttgen, which relies on FreeSurfer^40^ (v. 6) segmentation, applied to the T_1_-weighted (T_1_w) image.
- MS lesions: lesions were automatically segmented using the Lesion Prediction Algorithm (LPA)^41^ from the Lesion Segmentation Tool (LST) (v. 3.0.0) (www.statistical-modelling.de/lst.html), an open-source toolbox for Statistical Parametric Mapping (SPM) (v. 12). The toolbox generates an estimation of the lesion probability map, which was utilized to create a binary map of lesions. For each examination, the NAWM was identified by multiplying the inverse of this map with the WM mask. LST was applied only for patients’ exams; for controls, the NAWM corresponds to WM identified by MRtrix3.
- DTI regions: For each tract, seed and inclusion regions of interest for streamline generation and selection were defined on the Montreal Neurological Institute 152 (MNI152) standard brain. Non-linear registration between DWI and MNI spaces was performed using FNIRT from FSL^24^. Streamlines were generated using the iFOD1 method tckgen from MRtrix3^37^, inclusion regions, FOD amplitude, and deviation angle. The criteria used to define each tract are presented in Table 3 and Figs. 3 and 4. Tracts were individually reconstructed for the two hemispheres and then combined into a single region, converted into a NIfTI image with voxel intensity representing the streamline count. To obtain a relative fiber count estimate image, a threshold was applied (referring to the maximum value). Similar to NAWM, tract VOIs were analyzed excluding the lesions.

**Table 3:** Details for DTI implementation.

Tract	AF	CST	FAT	IFOF	OR	UF
**Seed**	Parietal lobe/peri-ventricular WM	Posterior Limb of Internal Capsule	Harvard-Oxford atlas supplementary motor area	Coronal plane at the anterior limit of the occipital lobe	Lateral geniculate nucleus	Superior temporal gyrus
**Obligatory waypoint(s)**	Frontal cortex and temporal cortex	Pons and precentral BN	Juelich atlas area BA44	Coronal plane, between the anterior limit of the genu of the corpus callosum and the anterior limit of the cingulate cortex	Visual cortex	VOI between insula and putamen
**Exclusion mask(s)**	Mid-sagittal plane	Mid-sagittal plane (*)	Mid-sagittal plane (*)	Mid-sagittal plane (*)	Mid-sagittal plane (*)	Mid-sagittal plane (*)
**Threshold (%)**	10	2	2	2	2	2

For each tract, seed, inclusion and exclusion regions for streamline generation and selection were defined on the MNI152 standard brain. In the table, details for each tract are summarized. The mid-sagittal plane was used as an exclusion mask for all the tracts, adding VOIs for some of them (*), specifically: squared VOI between the pons and middle cerebellar peduncles (axial/sagittal view) for CST; vertical plane at the anterior limit of the splenium of the corpus callosum, vertical plane at the anterior limit of the cingulate gyrus cortex, and VOI between insula and putamen for FAT; vertical plane anterior to mammillary bodies for IFOF; middle part of the pons/middle cerebellar peduncles (touching the posterior surface of the pons), WM/GM temporal lobe poles, under the superior temporal gyrus and laterally to uncus, mid-sagittal plane, superior to the pituitary gland and anterior to mammillary bodies and superior to posterior commissure/anterior to posterior limit of the splenium of the corpus callosum for OR; mid-coronal planes and lateral inferior limits of frontal lobes for UF. After the reconstruction, tracts were converted to a NIfTI image, and a minimum threshold was applied referring to the maximum value within the VOI. (AF = Arcuate Fasciculus, CST = Cortico-Spinal Tract, FAT = Frontal Aslant Tract, IFOF = Inferior Frontal-Occipital Fasciculus, OR = Optic Radiation, UF = Uncinate fasciculus, IS = inferior-superior, VOI = Volume of Interest, MNI152 = Montreal Neurological Institute’s 152).

### Feature extraction

PyRadiomics^42^ 3.0.1 (Python 3.7.6) was used to extract features from the VOIs overlain on the QSM images registered to the corresponding T_1_w space. VOIs were likewise registered to the T_1_w space, exploiting the previously obtained linear transformation matrices of FLAIR and DWI, using nearest neighbor interpolation to maintain binary masks. Since QSM and VOIs are in the same space, no additional interpolation operation was required for feature extraction, which was performed in 3D. MR exams were performed using the same clinical scanner and following the same acquisition protocol and processing pipeline, at the same center; this ensured sufficient homogeneity within the sample, obviating the need for histogram normalization steps.

Segment-based feature extraction was performed, meaning that each feature was extracted for each region of interest. Specifically, we considered 107 features, categorized as follows:

1. First-order features (FO, # 18): commonly used metrics to describe histogram intensity, including mean, median, 10^th^ and 90^th^ percentile, skewness, and kurtosis; FO measurements are independent of the number of Gray Levels (GLs);
2. Shape 3D features (S3D, # 14): descriptors of the 3D size and shape of the ROI (e.g., volume, surface, minimum and maximum axes); S3D measurements are independent of the number of GLs and their intensity distributions;
3. Gray Level Co-occurrence Matrix features (GLCM, # 24): describe the second-order joint probability function of an image region constrained by the mask;
4. Gray Level Run Length Matrix features (GLRLM, # 16): quantify GL runs (number of consecutive pixels that have the same grey level value);
5. Gray Level Size Zone Matrix features (GLZM, # 16): quantify GL zones in an image (number of the connected voxels that share the same grey level intensity);
6. Neighboring Gray Tone Difference Matrix features (NGTDM, # 5): quantify the difference between a GL value and the average grey value of its neighbors;
7. Gray Level Dependence Matrix features (GLDM, # 14): quantify GL dependencies in an image (number of connected voxels within a distance that are dependent on the central voxel).

Feature extraction was conducted with 64 as the number of gray levels (GLs), a parameter determined from the outcomes of a prior optimization study^23^. Categories 3 to 7 are referred to as texture and offer insights into the spatial distribution of intensity levels in the image. A complete list of the features can be found in the PyRadiomics documentation (https://pyradiomics.readthedocs.io/en/latest/features.html).

### Anonymization and de-identification

All images were anonymized and de-identified. The anonymization mask was created by combining: (1) the mask obtained processing T_1_w images with the automated defacing tools **mri_deface**^43^ from FreeSurfer^40^; (2) the mask obtained processing T_1_w images with SIENAX^44^ from FSL^24^; (3) the mask obtained with BET^29^ skull-stripping of the magnitude of the first echo time of the QSM sequence. Image files contain no metadata that can be used to identify study participants.

## Data Records

**Fig. 5:**
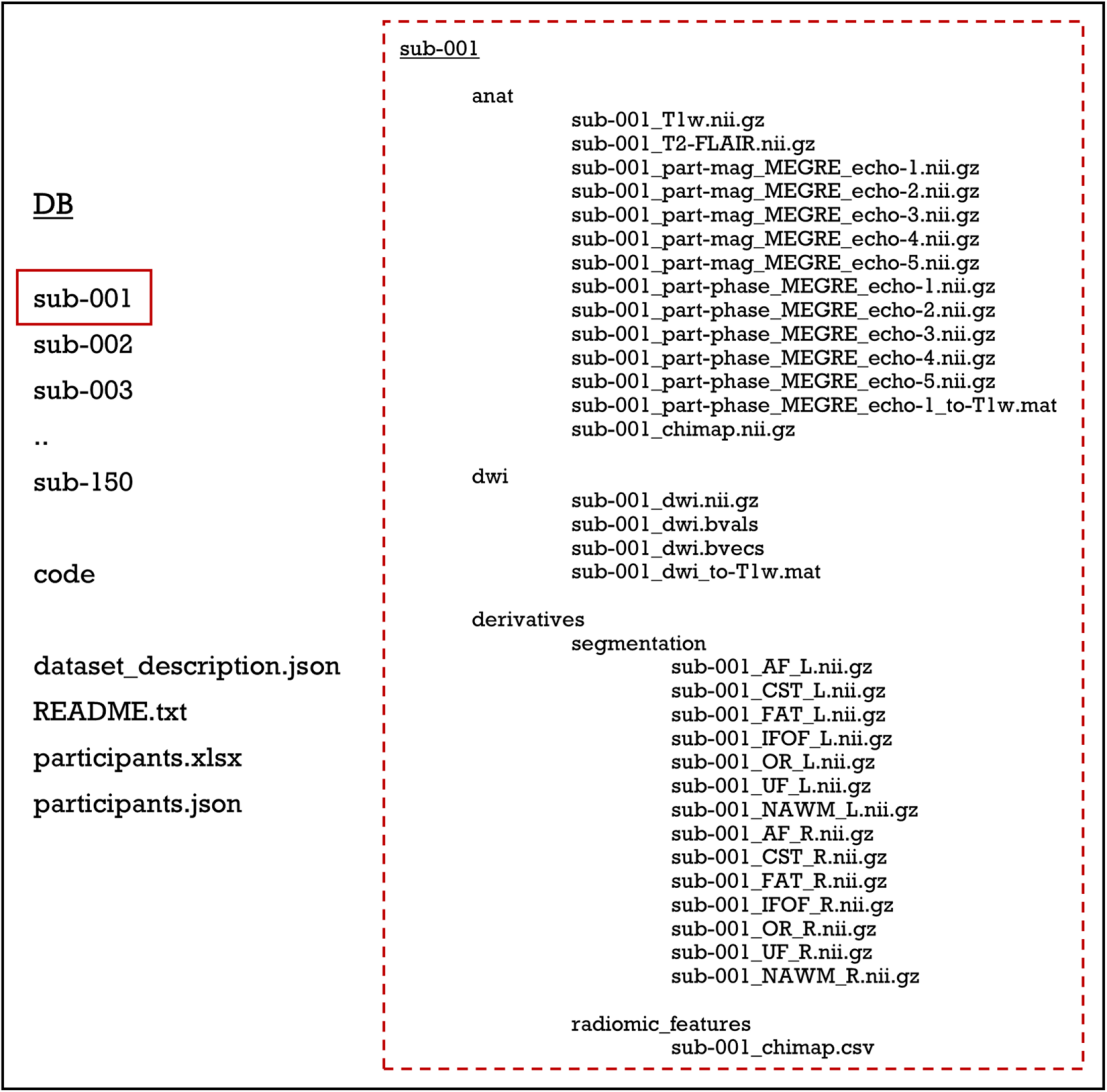
Schematic diagram of the dataset organization. A list of the participants to the dataset is provided and for each subject: anatomical T_1_w and T_2_w; DWI after the correction for EPI distortions and susceptibility effects, eddy currents and signal dropout, with b-values, b-vectors and the registration matrix to T_1_w; for QSM, original magnitude and phase maps for the five echo times, final QSM reconstruction registered in T_1_w space, with registration matrix to T_1_w; VOIs (AF, CST, FAT, IFOF, OR, UF) for left and right hemisphere;.csv files containing the 107 radiomic features for each region. Images were anonymized and de-identified (T_1_w = T_1_-weighted, T_2_w = T_2_-weighted, DWI = Diffusion-Weighted Imaging, QSM = Quantitative Susceptibility Mapping, VOI = Volume of Interest).

Files are organized according to the Brain Imaging Directory Structure (BIDS)^45^ (Fig. 5).

In the dataset, files are grouped in 150 folders, each corresponding to an individual subject, and organized as follows:

- In the ‘anat’ folder there are: a) T_1_-weighted (T_1_w), T_2_-FLAIR, and multi-echo GRE T_2_*w images (MEGRE) for QSM reconstruction, serving as non-structural MR images for each subject. For MEGRE, both phase and magnitude contributions are specified for each echo time; b) The QSM reconstruction, denoted with the suffix ‘chimap’, as a parametric non-structural image, along with the affine registration matrix to T_1_w. Specifically, the magnitude of the first echo time was used to register QSM to T_1_w. Given that orientation with respect to B_0_ is essential information for QSM processing, raw images are stored without registration, with the registration matrix to T_1_w provided separately, while the QSM reconstruction is already registered in T_1_w space.
- In the ‘dwi’ folder, there are: a) the diffusion weighted image after the correction for EPI distortions, susceptibility effects, eddy currents, and signal dropout, b) the required bvals and bvecs files, providing gradient orientation information corresponding to DWI volumes available (b = 0 and b = 2000 s/mm^2^), and c) the affine registration matrix to T_1_w.
- In the ‘derivatives’ folder there are the outputs from the processing pipeline. Two-subfolder were created: a) the segmentation folder, containing tracts and normal appearing white matter (NA-WM and tracts [AF, CST, FAT, IFOF, OR, UF] VOIs, divided for the two hemispheres) and b) the ‘radiomic_features’ folder, containing the.csv with all the features for each volume of interest (seven features for each hemisphere, resulting in a total of 14 features).
- Outside the folder of individual subjects, there is the ‘code’ folder containing the scripts used for processing tasks such as image registration, radiomic feature extraction, and robustness evaluation.
- Additional files are stored at the top level: a) dataset_description.json and README.txt files, describing the dataset; b) participants file (participants.xlsx and participants.json), containing the ID number, age, sex, scan date, clinical condition (denoted as ‘HC’ for healthy controls and ‘MS’ for multiple sclerosis patients), and the type of DWI sequence used (single-shell or multi-shell).

The size of the dataset^22^ is ~30GB and it is available the Zenodo repository.

## Usage Notes

To obtain access to the dataset^22^, the following steps must be followed:

1. An account must be created on the Zenodo repository, ensuring that either the username or the email address (or both) are public.
2. The Data Use Agreement, provided in the description of the dataset, must be completed, signed and send to radiomicsdataset@live.unibo.it.
3. Access will be granted within two working days, providing ‘Reader’ status to a community in which the dataset is included; the status can be monitored directly on Zenodo or via email.
4. Upon acceptance, access to the dataset will be granted, allowing for data download.

## Technical Validation

### Assessing robustness of susceptibility-based radiomic features

Hundreds of radiomic features can be extracted from MR images, enabling automated, high-throughput quantification of image characteristics^45^. This process provides a potentially extensive source of pathology-related biomarkers. It is essential to differentiate between image measurements and biomarkers^46^. Biomarkers are objective and quantifiable descriptors of biological processes capable of consistently predicting clinical outcomes and endpoints. Interpretability and reproducibility are two critical aspects in this context. In medical applications, it is crucial to maintain a clinical perspective as the driving force behind research^47^. The validity and relevance of a biomarker, along with its utility in clinical practice and its ability to offer valuable information, must be confirmed. Furthermore, when an informative and relevant feature is identified, it is vital to ensure the generalizability and replicability of outcomes. Since these features represent the outcome of physiological processes, they should remain consistent regardless of the imaging system or processing workflow used. The stages involved, from scanning to feature extraction, are numerous and complex. Thus, many parameters can impact the reliability of the results. In radiomic applications, robustness analysis is therefore essential to maintain the consistency of outcomes across different systems and multiple centers^45,48^.

In^23^, we describe a novel investigation into the robustness of susceptibility-based features derived from QSM. The study involved a cohort of 121 patients with MS and 30 healthy controls. We implemented an original and robust pipeline; we analyzed NAWM both as a whole and within the six clinically relevant tracts included in our dataset. To explore feature reliability, we varied the number of gray levels and echo times used for QSM reconstructions. After optimizing the number of GLs, set at n = 64, we found at least 65% of the features demonstrated robustness for each volume of interest. Notably, WM tracts exhibited higher levels of reliability, with over 75% of robust features in all of them. Differences among these tracts were explicable due to the volume of the structure and the susceptibility variance. No significant differences were observed between the left and right hemispheres.

The research confirmed the robustness of the data processing pipeline and established the reliability of QSM-based radiomic features against gray levels and echo times. This work paves the way for future investigations, where the identified set of reliable features may be used to characterize patients with MS, distinguish clinical phenotypes, and identify different responses to treatment.

## Data Availability

We hosted the code used for the study in the Zenodo repository^22^; the scripts provided can be used for image registration, radiomic feature extraction, and robustness analysis.
